# Metabolic reserves of diapausing western cherry fruit fly (Diptera: Tephritidae) pupae in relation to chill duration and post-chill rearing conditions

**DOI:** 10.3389/finsc.2022.989673

**Published:** 2022-09-16

**Authors:** Lisa G. Neven, Wee L. Yee

**Affiliations:** United States Department of Agriculture, Agricultural Research Service (USDA-ARS), Temperate Tree Fruit and Vegetable Research Unit, Wapato, WA, United States

**Keywords:** *Rhagoletis indifferens*, lipids, protein, carbohydrates, glycogen, diapause

## Abstract

How different macronutrients are utilized at various stages of pupal diapause and the effects of winter length on nutrient reserves remain poorly studied for most insects. Western cherry fruit fly, *Rhagoletis indifferens* (Diptera: Tephritidae), is a specialist on cherries in higher latitudes or elevations in western North America that exhibits a obligate pupal diapause requiring chilling before adult development can occur. We determined the relationship between metabolic reserves and diapause status in *R*. *indifferens* pupae, testing the hypotheses that lipids are the primary reserves utilized during diapause and that long periods of warmth deplete these reserves more than periods of cold. Effects of 0- to 20-week durations at 3°C and subsequent exposure to 23°C and 16:8 L:D (warm rearing conditions) for 0 to 7 weeks on lipid, protein, soluble carbohydrates, and glycogen reserves of *R*. *indifferens* pupae were determined. During diapause, lipid reserves were the primary source of energy utilized by *R. indifferens*, while protein and soluble carbohydrates levels were stable throughout diapause and thus less utilized. At post-diapause, glycogen levels fluctuated the most, indicating that lipid reserves were utilized to produce glycogen to support metabolism for adult fly development. Unchilled pupae did not deplete lipid reserves, unlike chilled pupae, likely because unchilled pupae remained in diapause. *Rhagoletis indifferens* may have evolved a nutrient utilization strategy typical of rigid diapausing insects in higher latitude environments.

## Introduction

Insects that undergo diapause to survive periods when food is unavailable or environmental conditions are unfavorable mitigate the energetic costs of diapause in part by accumulating energy reserves (1–5). Prior to diapause, macronutrients are accumulated in the form of triacylglyceride fat stores, the most important energy reserve in diapausing insects, carbohydrate reserve in the form of the polysaccharide glycogen, and protein (4). These macronutrients may not be utilized equally during development, as some insects switch from using lipids to non-lipid reserves at specific times during diapause (3).

Variation in macronutrient usage throughout diapause is common in Diptera. For example, in the flesh fly *Sarcophaga crassipalpis* Macquart (Diptera: Sarcophagidae), there is a shift from lipid dependence to protein and carbohydrate dependence over the course of diapause (6). *Rhagoletis cerasi* (L.) pupae (Diptera: Tephritidae) utilized lipid reserves during the early stages of pupal diapause, but over the winter switched to glycogen and carbohydrates as a metabolic resource (7). Despite the previous research on energy reserve utilization by Dipteran insects, how different macronutrients are utilized at various stages of pupal diapause remains poorly studied for most flies, including those that show differences in depth of diapause and whether it is obligate or facultative (8, 9).

Western cherry fruit fly, *Rhagoletis indifferens* Curran (Diptera: Tephritidae), is a temperate species native to western North America where it utilizes bitter cherry, *Prunus emarginata* (Dougl. ex Hook.) Eaton (ancestral host) as well as introduced cultivated sweet and tart cherries (*Prunus avium* L. and *Prunus cerasus* L., respectively) as hosts (10). The fly has an obligate seasonal pupal diapause in that less than 5% will eclose as adults within 2 to 7 weeks under warm conditions without cold, versus 55 to 90% in other *Rhagoletis* species that have a facultative diapause (8, 10–13). However, if unchilled pupae are held for an additional 27 to 40 weeks at warm temperatures, which would correlate to mid-winter and mid-spring, respectively, over 75% of those pupae will develop into adults (12, 13), suggesting a large portion of unchilled populations could diapause for 2 years.

The chill requirements needed by *R*. *indifferens* to synchronize adult eclosion have been studied in detail (10–13), in that pupae need to be exposed to temperatures at or below 3°C for a minimum of 140 to 200 days to complete diapause. However, there is a gap in knowledge of how pupae utilize nutrients during and after chill. It can be predicted from work on other insects (3, 4) that in *R*. *indifferens*, macronutrients in the form of lipids, protein, carbohydrates, and glycogen carried over from the feeding larval stage are differentially utilized during diapause and post diapause. In *R. cerasi*, diapausing pupae primarily utilize lipids to support metabolism during diapause while protein levels remained around 50 µg/mg fresh weight (7). Carbohydrate and glycogen levels tended to change more than protein levels especially at the beginning and end of diapause. Early fluctuations in glycogen could be related to the synthesis of polyhydroxy alcohols, like sorbitol and glycerol, to serve as cryoprotectants during exposure to low winter temperatures (7).

In addition to how macronutrients differ during pupal stages, another important question is how climate change that leads to shorter winter cold durations or warmer temperatures (14, 15) could affect energy reserves of fly pupae. In the U.S.A. and Canada, winter cold conditions have shortened by 15 d during the last 30 years (14). Fewer days of chilling may affect *R. indifferens* metabolic rate, diapause completion, and emergence synchronization thereby negatively impacting native populations. Previous research demonstrated that lack of sufficient chilling in central California may be an impediment to the establishment of *R. indifferens* (16). As climate change proceeds, the range of the fly may become more restricted as fewer and fewer areas experience low winter temperatures sufficient to trigger diapause completion. Additionally, the increasing soil temperatures in the summer might negatively impact *R*. *indifferens* pupal survival. Low winter temperatures suppress metabolism and conserve energy stores, so warmer winters might deplete reserves too early and jeopardize survival or post-diapause fitness (4). However, this hypothesis has not been tested for any *Rhagoletis* species and might depend on diapause traits of a species.

The objective of this study was to determine the relationship between metabolic reserves and diapause status in *R*. *indifferens* pupae, testing the hypotheses that (1) lipids are the primary reserves utilized during diapause and that (2) long periods of warmth deplete these reserves more than periods of cold. To this end, we specifically determined the metabolic reserves of diapausing *R. indifferens* in relation to early pupae after pupariation and pupae exposed to various durations of chilling followed by warm rearing conditions. We compare our results with those for other *Rhagoletis* species whose diapause traits differ from those of *R*. *indifferens*.

## Materials and methods

### Source of insects

*Rhagoletis indifferens*-infested sweet cherries were collected from unmanaged backyard trees of unidentified varieties in central Washington State in Benton, Franklin, Yakima, and Kennewick Counties at elevations of ~118-334 m over the 2014 field season. Cherries were collected on three dates in June and July 2014 for a total of three temporal harvest dates and five replicates for each treatment combination (12). Infested cherries were placed on wire mesh trays suspended above a soil mix spread over the bottom of a plastic tray. Puparia were collected daily for up to 7 d after cherry collections.

### Experimental design

Pupae from each harvest date were separated into treatments in groups of 25 for a total of 75 pupae per treatment. Pupae were conditioned at 23°C, 16:8 L:D and ~40% RH up to 15 d following collection. During this conditioning period, pupae were collected at 1, 2, 3, 5, 10, and 15 days after pupariation (early pupae) to determine initial biochemical levels. After the conditioning period, puparia were separated into treatment groups and held at 3°C ± 1.5°C for 0, 5, 10, and 20 weeks in an environmentally controlled cold room (Thermalrite, Plymouth, MN, U.S.A.) with a Chromalox (Ogden, UT, U.S.A.) controller. Pupae from each chill duration were then exposed to warm rearing conditions of 23°C, 16:8 L:D, and 40% RH for 0 to 7 weeks.

### Biochemical assays

Individual pupae, five for each chill + holding duration time point for each of the three harvest dates (total of 15 pupae for each), were weighed (XP205 analytical balance, Mettler Toledo, Columbus, OH, USA) and placed into a 1.5 ml microfuge tube. Only puparia weighing >5 mg were used for biochemical analyses since preliminary studies indicated that only puparia weighing >5 mg contained viable pupae (Neven, unpublished). Pupae were stored at -80°C until extractions for biochemical assays were performed. Total proteins, lipids, soluble carbohydrates, and glycogen were determined using a microtiter plate adaptation of methods published by Lee et al. (17), Olson et al. (18), van Handel (19), Yi and Jean (20), and Yuval et al. (21). Individual pupae were subjected to extraction using a 2% Na_2_SO_4_ solution and grinding with a pestle for 1 min. Ground samples were centrifuged for 1 min at 15,000 rpm (Eppendorf 5415R Benchtop Centrifuge, Germany) to pelletize the non-soluble insect body parts. The supernatant was used for determination of the biomolecules.

#### Proteins

An aliquot of the supernatant from the extraction tube was used to determine total proteins using the Bradford assay kit (Bio-Rad Protein Assay Kit II, #5000002, Bio-Rad Laboratories, Inc., Hercules, CA, USA) according to kit instructions. The remaining supernatant was subjected to chloroform:methanol (2:1) extraction to separate lipids from sugars and glycogen. After the addition of the chloroform:methanol solution, microfuge tubes were vortexed for 30 seconds and centrifuged for 4 min at 15,000 rpm. The solution was separated into layers, with sugars, glycogen, and lipids in the top, middle, and bottom layers, respectively. Each layer was individually removed and placed into individual microfuge tubes.

#### Lipids

The lipid supernatant was dried under a nitrogen stream. The lipid analysis was conducted in microtiter plates according to Yi and Jean (20), utilizing vanillin reagent. Each sample was replicated two times at three separate volumes (10, 20, and 50 µl). Standards were replicated three times on the plate. After the incubation period, the sample absorbance was read on a microplate reader (Varioskan, Thermo Electron Corporation) at 530 nM.

#### Sugars

Sugars were assessed using a modification of van Handel (19) and Lee et al. (17) utilizing anthrone reagent (anthrone in H_2_SO_4_). Standards were replicated three times on the microtiter plate and samples were replicated two times at three separate volumes (25, 50, and 75 µl). After the incubation period, the sample absorbance was read on a microplate reader at 620 nM.

#### Glycogen

The glycogen pellet, which was in the interface between the sugar and lipid layers, was assessed using a modification of the method by Lee et al. (17). The glycogen pellet was resolubilized in water prior to addition of the anthrone reagent. Standards were replicated three times on the microtiter plate and samples were replicated two times at three separate volumes (25, 50, and 75 µl). After the incubation period, the sample absorbance was read on a microplate reader at 620 nM.

### Statistics

Equality of variances were tested using SAS software and the generalized linear mixed models (GLM) procedure (22). All models resulted in convergence criteria being met for fixed effects. Datasets for the effects of cold duration and warm duration and effects of pupal age on the different biochemicals were analyzed using a 3-factor general linear model (PROC GLIMMIX) procedure (22). Fixed model interactions for Type 3 Tests of Fixed Effects were tested using the Kenward-Roger test. Data were plotted using Excel (Microsoft 365, 2020). The rates of lipid decrease in unchilled pupae and pupae receiving 20 weeks were determined using linear regression. To determine the relative usage of metabolic reserves, a MANOVA was performed using JMP (22, 23), with cold, warm, and harvest as independent variables and pupal weight, lipid content, protein content, soluble carbohydrates content, and glycogen content as dependent variables.

## Results

### Pupal weight

**Figure 1:**
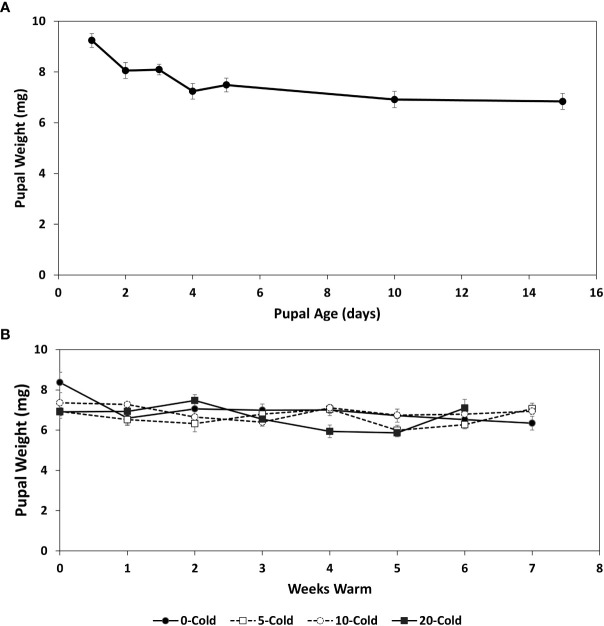
Pupal weights (mg ± SE) of *R. indifferens* from 1 to 15 days following pupation **(A)**, and after 0 to 20 weeks of chilling at 3°C, followed by 0 to 7 weeks at warm rearing conditions (23°C, 16:8 L:D, 40% RH) **(B)**. Each value is the average of five individuals from each of three different harvest dates.

**Figure 2:**
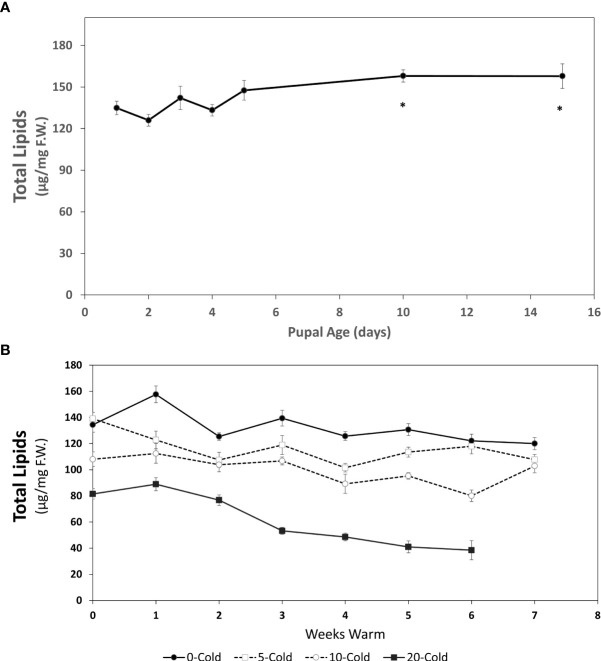
Total lipid content (µg/mg F.W. ± SE), of *R. indifferens* from 1 to 15 days following pupation **(A)**, and after 0 to 20 weeks of chilling at 3°C, followed by 0 to 7 weeks at warm rearing conditions (23°C, 16:8 L:D, 40% RH) **(B)**. Each value is the average of five individuals from each of the three different harvest dates.

**Figure 3:**
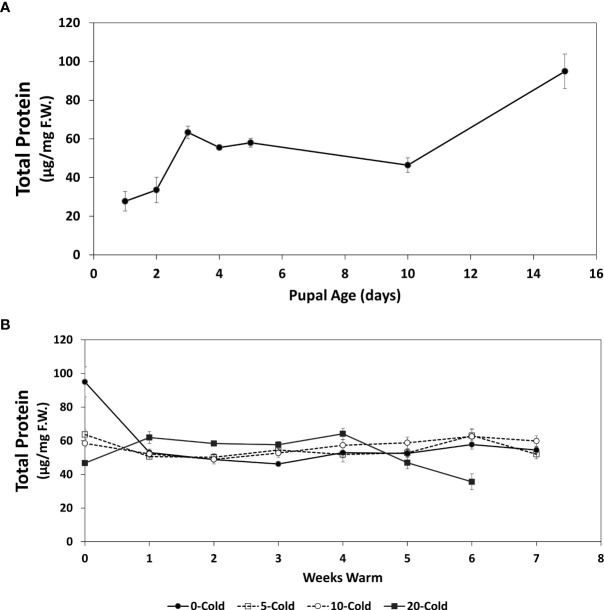
Total protein content (µg/mg F.W. ± SE), of *R. indifferens* from 1 to 15 days following pupation **(A)**, and after 0 to 20 weeks of chilling at 3°C, followed by 0 to 7 weeks at warm rearing conditions (23°C, 16:8 L:D, 40% RH) **(B)**. Each value is the average of five individuals from each of the three different harvest dates.

**Figure 4:**
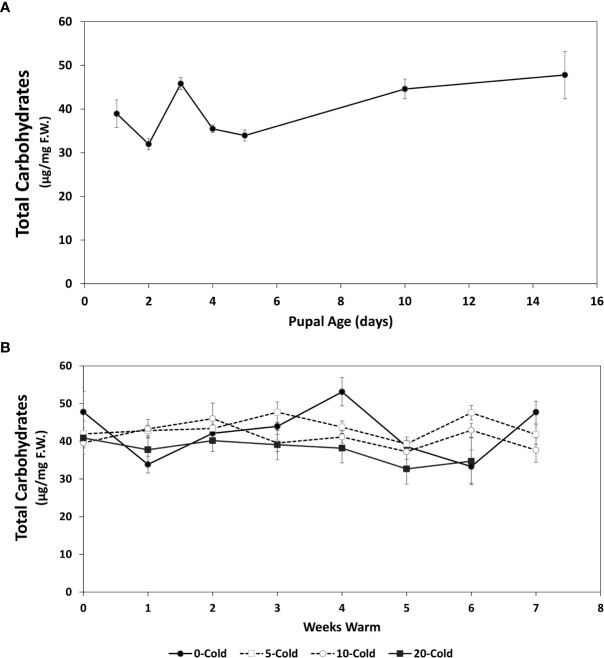
Total soluble carbohydrates content (µg/mg F.W. ± SE), of *R. indifferens* from 1 to 15 days following pupation **(A)**, and after 0 to 20 weeks of chilling at 3°C, followed by 0 to 7 weeks at warm rearing conditions (23°C, 16:8 L:D, 40% RH) **(B)**. Each value is the average of five individuals from each of the three different harvest dates.

**Figure 5:**
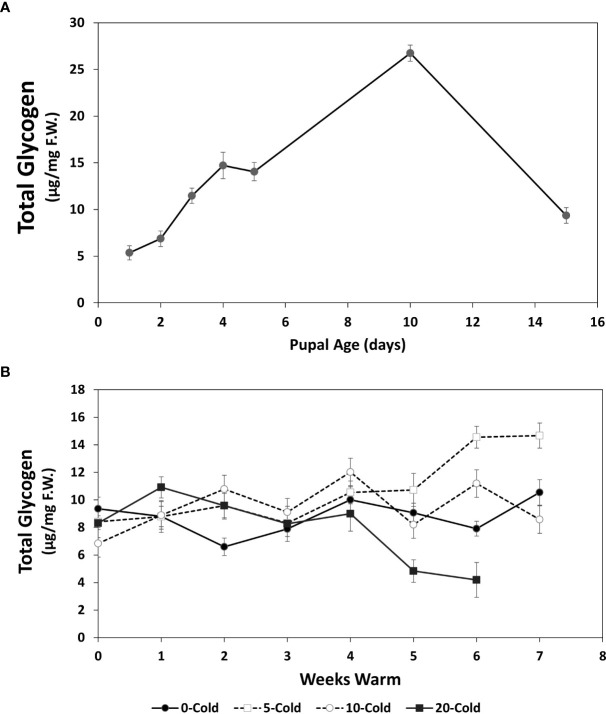
Total glycogen content (µg/mg F.W. ± SE), of *R. indifferens* from 1 to 15 days following pupation **(A)**, and after 0 to 20 weeks of chilling at 3°C, followed by 0 to 7 weeks at warm rearing conditions (23°C, 16:8 L:D, 40% RH) **(B)**. Each value is the average of five individuals from each of the three different harvest dates.

Pupal weights were highest at day one following pupariation, averaging 9.24 ( ± 0.26 SEM) mg, after which weights declined to 6.84 ± 1.22 mg by 15 days (**Figure 1A**). The pattern for pupal weight decrease, most likely an effect of desiccation, was reflected in the amounts of biochemicals, which generally increased in concentrations over the 15 day conditioning period (**Figures 2A**, **3A**, **4A**, **5A**). Mean pupal weights were stable throughout the cold storage and warm rearing conditions (**Figure 1B**), with no significant differences throughout the cold/warm durations (F_3,7_ = 2.182, *P*=0.503).

### Lipid content

A decrease in pupal weight (**Figure 1A**) coincided with an increase in total lipid content 0-5 days post pupariation and up to 15 days (early pupae) (**Figure 2A**). At day one following pupariation the average total lipid content was 134.88 ± 4.82 µg/mg F.W. [fresh weight] and increased to 157.85 ± 8.87 µg/mg F.W. (F_8,87 =_ 10.79, *P*<0.00014), an increase of 17.0%.

The total lipid content in pupae significantly decreased with longer cold duration (F_3,54 =_ 97.65, *P*<0.0001) and with more weeks at warm rearing conditions (F_7,54_ = 26.96, *P* < 0.0001) (**Figure 2B**). Unchilled pupae had the highest total lipid levels throughout the warm rearing duration. As the chilling duration increased, lipid levels decreased, with the lowest levels in pupae chilled for 20 weeks. Based on previous studies (13, 24) it is most likely that the 20 week chilled pupae had completed diapause and were in the process of completing development prior to eclosion. For all chilling durations, total lipid levels decreased as exposure to warm rearing conditions increased (**Table 1**).

**Table 1:** Results of MANOVA for lipid, protein, soluble carbohydrates, and glycogen content as dependent variables and Cold, Warm, and Cold : Warm interaction as independent variables * Statistically significant.

Whole Model					
*Test*	*Pillai*	*Approx F*	*Df*	*Den Df*	*Prob > F*
**Cold**	0.56606	136.314	1	418	<0.0001
**Warm**	0.16432	20.549	1	418	<0.0001
**Cold : Warm**	0.05595	6.193	1	418	<0.0001
**Residuals**			421		
** Lipid**	***Df***	***F***			***Prob > F***
**Cold**	1	527.191			<0.0001*
**Warm**	1	73.604			<0.0001*
**Cold : Warm**	1	10.257			<0.0001*
** Protein**	***Df***	***F***			***Prob > F***
**Cold**	1	3.366			0.0672
**Warm**	1	6.083			0.01404*
**Cold : Warm**	1	1.386			0.2397
** Soluble carbohydrates**	***Df***	***F***			***Prob > F***
**Cold**	1	9.1191			0.00268*
**Warm**	1	0.7348			0.39183
**Cold : Warm**	1	0.8483			0.35756
** Glycogen**		***F***			
**Cold**	1	1.8998			0.1688
**Warm**	1	2.5834			0.1087
**Cold : Warm**	1	9.5120			0.00218*

*Statistically significant.

Linear regression indicated that for unchilled pupae the slope for lipid decrease was -3.49 µg/mg·week^-1^ (R^2^ = 0.44; F_7_ = 4.63, P=0.04) while for 20-week chilled pupae it was -9.04 µg/mg·week^1^ (R^2^ = 0.89; F_7_ = 40.68, P= 0.014). Given that rate, unchilled pupae would deplete lipid reserves in approximately 41 weeks if held at 23°C, whereas pupae receiving 20 weeks of chilling would deplete lipid reserves in 9 weeks. Interestingly, these time points correlate with fly emergence peaks at 3-7 weeks and 27-40 weeks (13). The change in lipid content was the most pronounced in response to chilling conditions (**Table 1**) (F_1, 421_ = 527.191, *P* < 0.0001) and warm conditions (F_1, 421_ = 73.604, *P*< 0.0001), and less so with the combination of cold and warm conditions (F_1,421_ = 10.257, *P*=0.00146).

### Protein content

Similar to lipids (**Figure 2A**), total protein content in early pupae increased over the 15-day conditioning period (**Figure 3A**) (F_6,13_ = 3.75, *P* = 0.0218) coinciding with decreased pupal weight. One day and 15-day old pupae had average total protein contents of 27.71 ± 5.0 and 94.95 ± 8.91 µg/mg F.W., respectively, a 3.4-fold increase in total protein content.

The total protein content in pupae subjected to chilling and warm rearing conditions was stable after 1 week of warm rearing conditions, but there was a 42.6% drop in protein content in unchilled pupae from week 0 to week 1 at warm rearing conditions, from 94.95 ± 8.91 to 54.47 ± 1.79 µg/mg F.W. (F_1,60 _= 12.72, *P*<0.0001), respectively (**Figure 3B**). The lowest protein levels were recorded for pupae that had received 20 weeks of chilling and after 6 weeks of warm rearing at 35.02 ± 4.77 µg/mg F.W. MANOVA (**Table 1**) indicated that only warm duration caused significant changes in protein content (F_1,421_ = 6.0834, *P* = 0.01404).

### Soluble carbohydrate content

The total soluble carbohydrates increased in early pupae from 38.92 ± 3.15 µg/mg F.W.to 47.79 ± 5.43 as they aged from 1 to 15 days (F_6,11_ = 7.77, *P*=0.0017) (**Figure 4A**).

For pupae receiving chilling followed by warm rearing conditions, total soluble carbohydrate levels fluctuated around an average of 40 µg/mg among treatments (**Figure 4B**), but averages did not differ significantly (F_3,59_ = 2.54, *P*=0.065). However, soluble carbohydrate levels dropped significantly in relation to chilling duration (**Table 1**) (F _1,421_ = 9.1191, *P* = 0.0026).

### Glycogen content

Unlike the pattern seen with soluble carbohydrates, total glycogen levels showed a large increase in early pupae over the first 10 days following pupariation, with 1- to 10-day old pupae having averages of 5.35 ± 0.78 and 26.75 ± 0.85 µg/mg F.W., respectively (F_6, 11 =_ 7.77, *P*=0.0017) (**Figure 5A**). Notably, there was a 2.8-fold drop from day 10 to day 15 down to 9.36 ± 0.83 µg/mg F.W. In pupae that received chilling followed by warm rearing conditions, the changes in glycogen content varied from one another after 4 weeks of warm exposure (**Figure 5B**). Pupae that received no chilling had stable glycogen levels. Pupae that received 5 weeks of chilling showed the largest increase in total glycogen. Pupae that received 20 weeks of chilling showed the greatest drop in glycogen content over the warm rearing duration, from 8.57 ± 1.04 µg/mg F.W. at week 0, to 4.19 ± 1.26 at week 6, a 51.1% drop. Again, this indicated that this group had completed diapause and was continuing development to the adult stage. Total glycogen levels changed significantly in relation to the treatment of cold and warm duration (**Table 1**) (F_1_ = 9.5120, *P* =0.00218).

MANOVA of the pairwise comparisons of the biochemical level changes in response to the cold and warm durations indicated that there were no significant differences in relation to one another (**Table 1**).

## Discussion

This is the first report of metabolic reserve utilization by diapausing *R. indifferens*, a species with a high dependence on cold winters to terminate diapause (8). We found that *R. indifferens* utilizes lipid reserves during diapause. Glycogen levels varied the most in relation to the duration pupae were held under warm rearing conditions (23°C, 16:8 L:D, 40% RH), indicating the importance of this sugar to fuel diapause completion and post-diapause development.

The metabolic reserve use pattern of *R. indifferens* appears to differ slightly from that of *R. cerasi* in Europe (7), in that *R. cerasi* relies primarily on lipid and proteins as its major source of energy. *Rhagoletis cerasi* is an oligophagous, univoltine fly that exhibits a diapause similar to, but not the same as *R. indifferens* (9, 25). *Rhagoletis cerasi* may have a more complicated diapause strategy than *R*. *indifferens* in that it adapts to local ecological conditions while also responding plastically to unpredictable climatic conditions (7, 9, 26). Whether this occurs for *R. indifferens* remains to be tested.

In addition, based on predictive ecological models, *R*. *cerasi* may be capable of establishing in regions with relatively warm winters such as the San Joaquin Valley of California whereas *R*. *indifferens* cannot (27). After being chilled at 0, 5, 8, 10, or 12°C for 1-9 months, 40–55% of *R. cerasi* pupae remained dormant (9). In contrast, while not all *R*. *indifferens* produce adults after one winter, the proportion remaining dormant as pupae after 26 weeks was only 0.27% (10). However, Frick et al. (10) only held pupae for 26 weeks. Neven et al. (13) found that 75% of the unchilled pupae would produce adults if held under warm conditions for 40 weeks.

Two possible differences between nutrient utilization of *R*. *indifferens* and *R*. *cerasi* are important. *Rhagoletis cerasi* pupae utilize lipid reserves during the early stages of pupal diapause but switch to glycogen and carbohydrates over the winter (7). In contrast, while *R. indifferens* pupae appear to also use lipid during the early pupal stage, they did not have the decline in soluble carbohydrates observed for *R*. *cerasi*. Additionally, glycogen levels in *R. indifferens* were more variable than in *R*. *cerasi* throughout the warm rearing duration. Possibly the adaptation to local environments in western North America (*R. indifferens*) versus Europe (*R. cerasi*) may account for inter-species differences in nutrient utilization. Interestingly, *R. cerasi* exposed to >20 weeks at 5°C in laboratory tests to simulate highland environmental conditions (7) are more like our 20-week chilled pupae while *R*. *cerasi* exposed to simulated costal conditions are more like our 0- and 5- week chilled pupae. This suggests that environmental rather the genetic factors could play a large role in metabolic reserve utilization in both species.

The apple maggot fly, *Rhagoletis pomonella* (Walsh), is another *Rhagoletis* species whose diapause traits differ from those of *R*. *indifferens* (8), but to our knowledge there have been no studies on metabolic reserves of this fly. Unlike *R*. *indifferens*, subpopulations of *R. pomonella* do not require chill for adults to eclose and populations can exhibit no diapause, facultative diapause, and obligate diapause (28). Although metabolic reserves in *R. pomonella* throughout diapause and post-diapause development have not been reported, based on measurements of CO_2_ production (28), much of the energy metabolism also appears supported by lipids. However, metabolic reserves in non-diapausing phenotype of *R. pomonella* would be used up much faster than in diapausing individuals and could result in significant fitness cost to the individual (29). Despite the lack of nutrient reserve data for *R*. *pomonella*, the fact that *R. indifferens* utilizes lipid reserves during diapause and that its diapause, in general, lasts longer under identical conditions than of *R*. *pomonella* (24), it is possible that utilization of carbohydrates and glycogen fuel *R*. *pomonella* pupae over diapause and post-diapause development (30, 31).

Consistent with our findings of lipid utilization during diapause in *R*. *indifferens*, longer warm pre-chill and longer chill periods reduced post-diapause survival in *R. pomonella*, as chilling periods > 19 weeks probably depleted energy reserves of pupae below levels needed for morphogenesis (32, 33). However, given the differences in chill requirements between *R*. *indifferens* and *R*. *pomonella* (8), it is possible that longer chill durations would have a lesser negative effect on survival of *R*. *indifferens* than *R*. *pomonella*. This possibility warrants examination.

Contrary to the prediction that warmer winters could deplete reserves in diapausing insects too early (4), we found that lipid reserves of *R. indifferens* pupae held under warm conditions throughout (0 cold treatment, **Figure 2**) were not depleted versus those of pupae chilled for 5-20 weeks (**Figure 2**). Protein, carbohydrate, and glycogen levels were also not affected by no-chill. Irwin and Lee (34) reported that fewer adults of goldenrod gall fly *Eurosta solidaginis* (Fitch) (Diptera: Tephritidae) emerged from the warmer than colder galls, attributable to reduced reserves under warmer conditions. Although we noted a reduction in adult emergence in unchilled *R. indifferens*, we posit that critical metabolic reserves are not utilized by pupae remaining in diapause with very low metabolic rates (Neven, unpublished) (13). In the blueberry bee *Osmia ribifloris* Cockerell (Hymenoptera: Megachilidae), which undergoes a period of prepupal diapause and adult diapause, a warming treatment resulted in reduced body mass and fat content and increased mortality (35). Warm temperatures accelerated carbohydrate and protein but not lipid utilization in diapausing pupae of the fall webworm *Hyphantria cunea* (Drury) (Lepidoptera: Erebidae) (36).

The lack of lipid and other nutrient depletion in *R. indifferens* pupae that were never chilled could be related to lack of post-diapause development. Pupae receiving the required duration of chilling to complete diapause proceeded with development and thus needed to utilize nutrients for morphogenesis. This is consistent with the hypothesis that longer chill durations (20 versus 10 versus 5 weeks) for *R. indifferens* stimulate more rapid fly development, as measured by timing of fly eclosion (11, 13), after transfer to warm conditions. Faster development should result in greater energy expenditure per unit time.

The higher lipid reserves in *R*. *indifferens* pupae in the no-chill treatment may appear beneficial, allowing for prolonged survival, and perhaps a longer diapause into a second season of cooler winter temperatures needed to trigger the completion of diapause and continuation of pupal development and adult eclosion. Thus, the higher or at least stable nutrient reserves due to no or shorter winter cold confer no benefit to a rigid diapausing insect such as *R*. *indifferens*. In contrast, for multivoltine or facultative diapausing species, there may be a benefit of a warming climate. For the multivoltine blow fly *Calliphora vicina* Robineau-Desvoidy (Diptera: Calliphoridae), warmer winters corresponded to shorter larval diapause, enabling the fly to carry over more of its larval reserves into adulthood, perhaps enhancing fecundity (37). For insects with facultative diapause, a decrease in winter duration can add one or more generations to their seasonal cycle (38 and references therein). Although it is unclear if increased voltinism always results in increased fitness, it can be assumed that it directly is related to utilization of energy reserves.

*Rhagoletis indifferens* may have evolved a strategy of nutrient utilization typical of rigid diapausing insects in higher latitude environments, a hypothesis that needs to be tested by studying more insect species, for both chill and no-chill effects. Such species could include *Rhagoletis tabellaria* (Fitch) and *Rhagoletis zephyria* Snow, two flies that occupy the same habitats as *R. indifferens* and that also have rigid winter diapause (39, 40). These species can then be compared with *R. pomonella* and the walnut husk fly, *Rhagoletis completa* Cresson, species with a very different, strongly facultative diapause (8). Costs of diapause on metabolic reserves and fitness vary greatly among insects (3). Thus, such comparative studies should aim to discover general patterns in nutrient utilization of flies with very specific diapause requirements, resulting in principles that can be applied to a broad range of insects.

## Data availability statement

The raw data supporting the conclusions of this article will be made available by the authors, without undue reservation.

## Author contributions

Both authors contributed equally to the development, execution, data analysis, and writing of the manuscript.

## Funding

This research was funded by a grant from the Washington Tree Fruit Commission and the USDA-FAS-TASC program. USDA is an equal opportunity provider and employer. Agreement Number 58-5352-1-192.

## Acknowledgments

The authors would like to thank Jennifer Stout, Michele Watkins, Anne Kenny Chapman, Linda Rehfield-Ray, Kelly Thomason-Archer, Jenny Harris, Dana Jones, and Emily Call of USDA-ARS-TTFVRU for their technical assistance. We also thank Nathan Lehrman, Central Washington University Masters student for his assistance in the determination of biochemical reserves. We would like to thank Dr. Gina Angelella of USDAARS-TTFVRU for her assistance with MANOVA analyses.

## Conflict of interest

The authors declare that the research was conducted in the absence of any commercial or financial relationships that could be construed as a potential conflict of interest.

## Publisher’s note

All claims expressed in this article are solely those of the authors and do not necessarily represent those of their affiliated organizations, or those of the publisher, the editors and the reviewers. Any product that may be evaluated in this article, or claim that may be made by its manufacturer, is not guaranteed or endorsed by the publisher.
